# 1-Phenyl-5-[4-(trifluoro­meth­yl)phen­yl]­pyrazolidin-3-one monohydrate

**DOI:** 10.1107/S1600536808032261

**Published:** 2008-10-11

**Authors:** Yuan-Yuan Liu, Zhen-Yi Wu, Hong Shi, Qing-Yan Chu, Hong-Jun Zhu

**Affiliations:** a Department of Applied Chemistry, College of Science, Nanjing University of Technology, Nanjing 210009, People’s Republic of China

## Abstract

In the mol­ecule of the title compound, C_16_H_13_F_3_N_2_O·H_2_O, the two benzene rings are oriented at a dihedral angle of 82.55 (3)° and the pyrazole ring adopts an envelope conformation. In the crystal structure, inter­molecular C—H⋯F hydrogen bonds link the mol­ecules into chains.

## Related literature

For general background, see: Menozzi *et al.* (1990 ▶); James & William (2003 ▶); Shi *et al.* (2006 ▶). For related literature, see: Jia *et al.* (2008 ▶). For bond-length data, see: Allen *et al.* (1987 ▶).
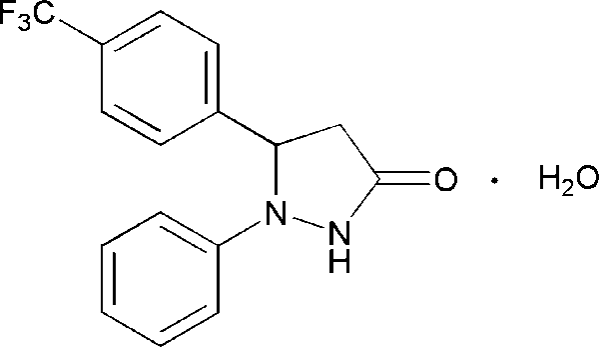


## Experimental

### 

#### Crystal data

C_16_H_13_F_3_N_2_O·H_2_O
*M* *_r_* = 324.30
Triclinic, 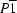

*a* = 7.4960 (15) Å
*b* = 9.794 (2) Å
*c* = 13.319 (3) Å
α = 97.70 (3)°
β = 101.58 (3)°
γ = 107.97 (3)°
*V* = 890.8 (4) Å^3^
*Z* = 2
Mo *K*α radiation
μ = 0.10 mm^−1^
*T* = 294 (2) K
0.20 × 0.10 × 0.05 mm

#### Data collection

Enraf–Nonius CAD-4 diffractometer
Absorption correction: ψ scan (North *et al.*, 1968 ▶) *T*_min_ = 0.980, *T*_max_ = 0.995
3463 measured reflections
3196 independent reflections
1611 reflections with *I* > 2σ(*I*)
*R*_int_ = 0.068
3 standard reflections frequency: 120 min intensity decay: none

#### Refinement

*R*[*F* ^2^ > 2σ(*F* ^2^)] = 0.077
*wR*(*F* ^2^) = 0.186
*S* = 1.00
3196 reflections
211 parameters
48 restraints
H-atom parameters constrained
Δρ_max_ = 0.21 e Å^−3^
Δρ_min_ = −0.23 e Å^−3^

### 

Data collection: *CAD-4 Software* (Enraf–Nonius, 1985 ▶); cell refinement: *CAD-4 Software*; data reduction: *XCAD4* (Harms & Wocadlo, 1995 ▶); program(s) used to solve structure: *SHELXS97* (Sheldrick, 2008 ▶); program(s) used to refine structure: *SHELXL97* (Sheldrick, 2008 ▶); molecular graphics: *SHELXTL* (Sheldrick, 2008 ▶); software used to prepare material for publication: *SHELXTL*.

## Supplementary Material

Crystal structure: contains datablocks I, global. DOI: 10.1107/S1600536808032261/hk2518sup1.cif

Structure factors: contains datablocks I. DOI: 10.1107/S1600536808032261/hk2518Isup2.hkl

Additional supplementary materials:  crystallographic information; 3D view; checkCIF report

## Figures and Tables

**Table 1:** Hydrogen-bond geometry (Å, °)

*D*—H⋯*A*	*D*—H	H⋯*A*	*D*⋯*A*	*D*—H⋯*A*
O*W*2—H*W*2*B*⋯O*W*1	0.85	1.99	2.50 (3)	118
C14—H14*A*⋯F3^i^	0.93	2.54	3.262 (6)	134
N1—H1*A*⋯O^ii^	0.86	1.98	2.811 (6)	161
C9—H9*B*⋯O^iii^	0.97	2.60	3.555 (7)	168

Symmetry code: (i) 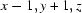; (ii) 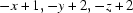; (iii) 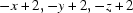.

**Fig. 1.**
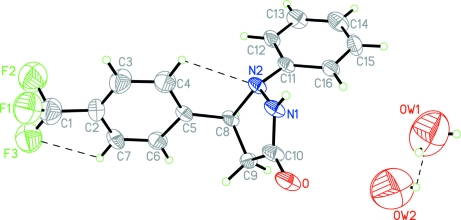
The molecular structure of the title molecule, with the atom-numbering scheme. Displacement ellipsoids are drawn at the 50% probability level. Hydrogen bonds are shown as dotted lines.

**Fig. 2.**
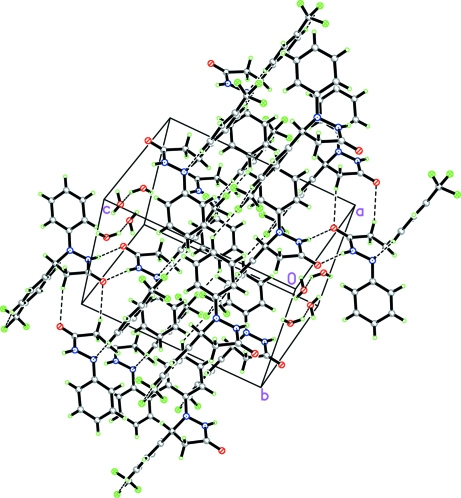
A packing diagram of the title compound. Hydrogen bonds are shown as dashed lines.

## supplementary crystallographic information

### Comment

Nowadays, heterocyclic compounds as medicines and pesticides have been developed most quickly. Among them, pyrazole and its derivatives exhibit better bioactivity, such as antipyretic (Menozzi *et al.*,  1990) and anticancer (James & William, 2003) activities. They are also useful in the treatment of inflammation and related disorders (Shi *et al.*, 2006). We report herein the crystal structure of the title compound.

In the molecule of the title compound (Fig. 1), the bond lengths (Allen *et al.*,  1987) and angles are within normal ranges. Rings A (C2-C7) and C (C11-C16) are, of course, planar and the dihedral angle between them is 82.55 (3)°. Ring B (N1/N2/C8-C10) is not planar, and adopts envelope conformation with C8 atom displaced by 0.358 (3) Å from the plane of the other ring atoms. The intramolecular C-H···N and C-H···F hydrogen bonds (Table 1) result in the formation of two planar five-membered rings D (N2/C4/C5/C8/H4A) and E (F3/C1/C2/C7/H7A). They are oriented with respect to ring A at dihedral angles of 3.20 (3)° and 2.57 (3)°, respectively. So, rings A, D and E are nearly coplanar.

In the crystal structure, intermolecular C-H···F hydrogen bonds (Table 1) link the molecules into chains (Fig. 2), in which they may be effective in the stabilization of the structure.

### Experimental

The title compound was prepared by the literature method (Jia *et al.*, 2008). For the preparation of the title compound, n-butanol (40 ml) and ethanolamine (4 ml) were added to a solution of sodium (40 mmol) in anhydrous methanol (9 ml). Then, methanol was removed by distillation and methyl 3-(4-(trifluoromethyl)phenyl)acrylate (30 mmol) was added. The mixture was refluxed for 1.5 h at 385 K, after which phenylhydrazine (4 ml) was added. The mixture was refluxed for another 10 h, and then left to cool to room temperature. It was then acidified with acetic acid (50%), allowed to stand and filtered. The filter cake was chromatographed over silica gel (500 g) eluting with a mixture of ethyl acetate and petroleum ether to give the title compound (m.p. 413- 415 K). Crystals suitable for x-ray analysis were obtained by dissolving the title compound (1.5 g) in ethyl acetate (25 ml) and evaporating the solvent slowly at room temperature for about 10 d.

### Refinement

The water molecule was disordered. During the refinement process the disordered O and H atoms were refined with occupancies of 0.50. H atoms were positioned geometrically, with O-H = 0.85 Å (for H_2_O), N-H = 0.86 (for NH) and C-H = 0.93, 0.98 and 0.97 Å for aromatic, methine and methylene H, respectively, and constrained to ride on their parent atoms with U_iso_(H) = 1.2U_eq_(C,N,O).

### Crystal data

**Table:**

C_16_H_13_F_3_N_2_O·H_2_O	*Z* = 2
*M**_r_* = 324.30	*F*(000) = 336
Triclinic, *P*1	*D*_x_ = 1.209 Mg m^−^^3^
Hall symbol: -P 1	Mo *K*α radiation, λ = 0.71073 Å
*a* = 7.4960 (15) Å	Cell parameters from 25 reflections
*b* = 9.794 (2) Å	θ = 9–12°
*c* = 13.319 (3) Å	µ = 0.10 mm^−^^1^
α = 97.70 (3)°	*T* = 294 K
β = 101.58 (3)°	Needle, colorless
γ = 107.97 (3)°	0.20 × 0.10 × 0.05 mm
*V* = 890.8 (4) Å^3^	

### Data collection

**Table:**

Enraf–Nonius CAD-4 diffractometer	1611 reflections with *I* > 2σ(*I*)
Radiation source: fine-focus sealed tube	*R*_int_ = 0.068
graphite	θ_max_ = 25.2°, θ_min_ = 1.6°
ω/2θ scans	*h* = −8→8
Absorption correction: ψ scan (North *et al.*, 1968)	*k* = −11→11
*T*_min_ = 0.980, *T*_max_ = 0.995	*l* = 0→15
3463 measured reflections	3 standard reflections every 120 min
3196 independent reflections	intensity decay: none

### Refinement

**Table:**

Refinement on *F*^2^	Primary atom site location: structure-invariant direct methods
Least-squares matrix: full	Secondary atom site location: difference Fourier map
*R*[*F*^2^ > 2σ(*F*^2^)] = 0.077	Hydrogen site location: inferred from neighbouring sites
*wR*(*F*^2^) = 0.186	H-atom parameters constrained
*S* = 1.00	*w* = 1/[σ^2^(*F*_o_^2^) + (0.06*P*)^2^ + 0.58*P*] where *P* = (*F*_o_^2^ + 2*F*_c_^2^)/3
3196 reflections	(Δ/σ)_max_ < 0.001
211 parameters	Δρ_max_ = 0.21 e Å^−^^3^
48 restraints	Δρ_min_ = −0.23 e Å^−^^3^

### Special details

**Table:**

Geometry. All e.s.d.'s (except the e.s.d. in the dihedral angle between two l.s. planes) are estimated using the full covariance matrix. The cell e.s.d.'s are taken into account individually in the estimation of e.s.d.'s in distances, angles and torsion angles; correlations between e.s.d.'s in cell parameters are only used when they are defined by crystal symmetry. An approximate (isotropic) treatment of cell e.s.d.'s is used for estimating e.s.d.'s involving l.s. planes.
Refinement. Refinement of *F*^2^ against ALL reflections. The weighted *R*-factor *wR* and goodness of fit *S* are based on *F*^2^, conventional *R*-factors *R* are based on *F*, with *F* set to zero for negative *F*^2^. The threshold expression of *F*^2^ > σ(*F*^2^) is used only for calculating *R*-factors(gt) *etc*. and is not relevant to the choice of reflections for refinement. *R*-factors based on *F*^2^ are statistically about twice as large as those based on *F*, and *R*- factors based on ALL data will be even larger.

### Fractional atomic coordinates and isotropic or equivalent isotropic displacement parameters (Å^2^)

**Table:**

	*x*	*y*	*z*	*U*_iso_*/*U*_eq_	Occ. (<1)
OW1	0.464 (3)	1.4138 (15)	1.0235 (11)	0.288 (8)	0.50
HW1A	0.5305	1.3813	1.0675	0.345*	0.50
HW1B	0.4367	1.4891	1.0439	0.345*	0.50
OW2	0.805 (3)	1.5022 (19)	1.0058 (13)	0.328 (9)	0.50
HW2A	0.8673	1.5642	0.9752	0.394*	0.50
HW2B	0.7436	1.5280	1.0477	0.394*	0.50
O	0.7711 (4)	1.0639 (3)	1.0297 (2)	0.0741 (9)	
N1	0.5378 (4)	0.9682 (3)	0.8750 (2)	0.0520 (8)	
H1A	0.4422	0.9388	0.9027	0.062*	
N2	0.5122 (4)	0.9482 (3)	0.7661 (2)	0.0495 (7)	
F1	0.8128 (5)	0.3357 (4)	0.6898 (3)	0.1429 (12)	
F2	0.7729 (6)	0.3635 (4)	0.5389 (3)	0.1549 (14)	
F3	1.0292 (5)	0.4452 (4)	0.6316 (3)	0.1343 (11)	
C1	0.8642 (9)	0.4424 (7)	0.6409 (5)	0.097	
C2	0.8153 (7)	0.5692 (4)	0.6625 (3)	0.0695 (11)	
C3	0.6372 (7)	0.5629 (5)	0.6753 (4)	0.0924 (15)	
H3A	0.5419	0.4721	0.6676	0.111*	
C4	0.5972 (6)	0.6888 (5)	0.6994 (4)	0.0869 (14)	
H4A	0.4717	0.6827	0.7014	0.104*	
C5	0.7395 (5)	0.8228 (4)	0.7207 (3)	0.0480 (9)	
C6	0.9191 (5)	0.8233 (4)	0.7059 (3)	0.0654 (11)	
H6A	1.0176	0.9128	0.7150	0.079*	
C7	0.9539 (6)	0.6985 (5)	0.6791 (3)	0.0660 (11)	
H7A	1.0764	0.7030	0.6721	0.079*	
C8	0.7150 (4)	0.9668 (4)	0.7541 (2)	0.0443 (8)	
H8A	0.7384	1.0243	0.7003	0.053*	
C9	0.8485 (5)	1.0570 (4)	0.8573 (2)	0.0512 (9)	
H9A	0.9001	1.1596	0.8538	0.061*	
H9B	0.9556	1.0222	0.8777	0.061*	
C10	0.7225 (5)	1.0368 (4)	0.9337 (3)	0.0568 (10)	
C11	0.4190 (4)	1.0326 (4)	0.7171 (3)	0.0485 (9)	
C12	0.3624 (5)	1.0040 (4)	0.6080 (3)	0.0587 (10)	
H12A	0.3921	0.9310	0.5698	0.070*	
C13	0.2660 (6)	1.0793 (5)	0.5571 (3)	0.0694 (12)	
H13A	0.2318	1.0592	0.4842	0.083*	
C14	0.2170 (6)	1.1866 (5)	0.6116 (4)	0.0759 (12)	
H14A	0.1510	1.2393	0.5760	0.091*	
C15	0.2666 (6)	1.2142 (4)	0.7181 (4)	0.0716 (12)	
H15A	0.2292	1.2840	0.7545	0.086*	
C16	0.3695 (5)	1.1430 (4)	0.7737 (3)	0.0557 (10)	
H16A	0.4060	1.1662	0.8466	0.067*	

### Atomic displacement parameters (Å^2^)

**Table:**

	*U*^11^	*U*^22^	*U*^33^	*U*^12^	*U*^13^	*U*^23^
OW1	0.37 (2)	0.239 (16)	0.249 (16)	0.119 (17)	0.069 (15)	0.038 (12)
OW2	0.31 (2)	0.312 (19)	0.33 (2)	0.089 (17)	0.053 (17)	0.039 (15)
O	0.0489 (17)	0.132 (3)	0.0524 (17)	0.0415 (16)	0.0149 (13)	0.0292 (16)
N1	0.0365 (17)	0.082 (2)	0.0465 (17)	0.0262 (16)	0.0146 (14)	0.0257 (15)
N2	0.0361 (16)	0.0695 (19)	0.0517 (18)	0.0246 (15)	0.0166 (14)	0.0191 (15)
F1	0.161 (3)	0.128 (3)	0.146 (3)	0.051 (2)	0.048 (3)	0.037 (2)
F2	0.168 (4)	0.152 (3)	0.143 (3)	0.061 (3)	0.033 (3)	0.020 (2)
F3	0.134 (3)	0.131 (3)	0.149 (3)	0.054 (2)	0.046 (2)	0.030 (2)
C1	0.106	0.099	0.095	0.045	0.038	0.010
C2	0.080 (3)	0.060 (2)	0.079 (3)	0.037 (2)	0.025 (2)	0.013 (2)
C3	0.084 (3)	0.070 (3)	0.119 (4)	0.013 (2)	0.043 (3)	0.009 (3)
C4	0.053 (3)	0.076 (3)	0.124 (4)	0.011 (2)	0.037 (3)	0.004 (3)
C5	0.0356 (19)	0.057 (2)	0.056 (2)	0.0196 (17)	0.0133 (16)	0.0184 (17)
C6	0.039 (2)	0.071 (2)	0.083 (3)	0.0130 (19)	0.023 (2)	0.007 (2)
C7	0.053 (2)	0.078 (3)	0.082 (3)	0.036 (2)	0.026 (2)	0.022 (2)
C8	0.0291 (18)	0.068 (2)	0.0437 (19)	0.0187 (17)	0.0230 (15)	0.0132 (17)
C9	0.0347 (19)	0.070 (2)	0.046 (2)	0.0167 (18)	0.0150 (16)	−0.0019 (17)
C10	0.036 (2)	0.087 (3)	0.053 (2)	0.021 (2)	0.0159 (18)	0.026 (2)
C11	0.0298 (19)	0.064 (2)	0.058 (2)	0.0191 (17)	0.0170 (17)	0.0159 (18)
C12	0.054 (2)	0.088 (3)	0.043 (2)	0.040 (2)	0.0123 (18)	0.0052 (19)
C13	0.069 (3)	0.110 (3)	0.040 (2)	0.039 (3)	0.018 (2)	0.025 (2)
C14	0.065 (3)	0.104 (3)	0.074 (3)	0.043 (3)	0.019 (2)	0.039 (3)
C15	0.065 (3)	0.069 (3)	0.095 (3)	0.040 (2)	0.029 (2)	0.012 (2)
C16	0.044 (2)	0.072 (2)	0.053 (2)	0.025 (2)	0.0106 (18)	0.0093 (19)

### Geometric parameters (Å, °)

**Table:**

OW1—HW1A	0.8502	C5—C8	1.499 (4)
OW1—HW1B	0.8499	C6—C7	1.344 (5)
OW2—HW2A	0.8500	C6—H6A	0.9300
OW2—HW2B	0.8499	C7—H7A	0.9300
O—C10	1.226 (4)	C8—C9	1.499 (4)
N1—C10	1.352 (4)	C8—H8A	0.9800
N1—N2	1.404 (4)	C9—C10	1.513 (4)
N1—H1A	0.8600	C9—H9A	0.9700
N2—C11	1.384 (4)	C9—H9B	0.9700
N2—C8	1.518 (4)	C11—C12	1.392 (5)
F1—C1	1.303 (6)	C11—C16	1.420 (5)
F2—C1	1.389 (6)	C12—C13	1.339 (5)
F3—C1	1.260 (6)	C12—H12A	0.9300
C1—C2	1.409 (6)	C13—C14	1.381 (5)
C2—C7	1.324 (5)	C13—H13A	0.9300
C2—C3	1.363 (6)	C14—C15	1.358 (5)
C3—C4	1.369 (6)	C14—H14A	0.9300
C3—H3A	0.9300	C15—C16	1.368 (5)
C4—C5	1.363 (5)	C15—H15A	0.9300
C4—H4A	0.9300	C16—H16A	0.9300
C5—C6	1.398 (4)		
			
HW1A—OW1—HW1B	120.0	C5—C8—N2	112.4 (3)
HW2A—OW2—HW2B	120.0	C9—C8—N2	105.1 (2)
C10—N1—N2	115.7 (3)	C5—C8—H8A	108.2
C10—N1—H1A	122.2	C9—C8—H8A	108.2
N2—N1—H1A	122.2	N2—C8—H8A	108.2
C11—N2—N1	115.6 (3)	C8—C9—C10	104.5 (3)
C11—N2—C8	116.8 (3)	C8—C9—H9A	110.9
N1—N2—C8	102.8 (2)	C10—C9—H9A	110.9
F3—C1—F1	103.2 (5)	C8—C9—H9B	110.9
F3—C1—F2	92.8 (4)	C10—C9—H9B	110.9
F1—C1—F2	98.4 (5)	H9A—C9—H9B	108.9
F3—C1—C2	123.8 (6)	O—C10—N1	124.4 (3)
F1—C1—C2	120.7 (5)	O—C10—C9	129.1 (3)
F2—C1—C2	111.8 (5)	N1—C10—C9	106.2 (3)
C7—C2—C3	119.5 (4)	N2—C11—C12	119.1 (3)
C7—C2—C1	117.8 (5)	N2—C11—C16	122.5 (3)
C3—C2—C1	122.4 (5)	C12—C11—C16	118.3 (3)
C2—C3—C4	120.7 (4)	C13—C12—C11	121.3 (3)
C2—C3—H3A	119.7	C13—C12—H12A	119.3
C4—C3—H3A	119.7	C11—C12—H12A	119.3
C5—C4—C3	120.7 (4)	C12—C13—C14	120.7 (4)
C5—C4—H4A	119.7	C12—C13—H13A	119.6
C3—C4—H4A	119.7	C14—C13—H13A	119.6
C4—C5—C6	116.1 (4)	C15—C14—C13	119.0 (4)
C4—C5—C8	125.2 (3)	C15—C14—H14A	120.5
C6—C5—C8	118.7 (3)	C13—C14—H14A	120.5
C7—C6—C5	122.1 (4)	C14—C15—C16	122.4 (4)
C7—C6—H6A	118.9	C14—C15—H15A	118.8
C5—C6—H6A	118.9	C16—C15—H15A	118.8
C2—C7—C6	120.6 (4)	C15—C16—C11	118.1 (3)
C2—C7—H7A	119.7	C15—C16—H16A	120.9
C6—C7—H7A	119.7	C11—C16—H16A	120.9
C5—C8—C9	114.7 (3)		
			
C10—N1—N2—C11	−109.8 (3)	C11—N2—C8—C5	−130.5 (3)
C10—N1—N2—C8	18.6 (4)	N1—N2—C8—C5	101.8 (3)
F3—C1—C2—C7	−3.6 (8)	C11—N2—C8—C9	104.2 (3)
F1—C1—C2—C7	132.2 (6)	N1—N2—C8—C9	−23.5 (3)
F2—C1—C2—C7	−112.9 (5)	C5—C8—C9—C10	−103.1 (3)
F3—C1—C2—C3	−178.0 (5)	N2—C8—C9—C10	20.8 (4)
F1—C1—C2—C3	−42.2 (8)	N2—N1—C10—O	179.7 (3)
F2—C1—C2—C3	72.7 (7)	N2—N1—C10—C9	−5.5 (4)
C7—C2—C3—C4	3.6 (7)	C8—C9—C10—O	164.2 (4)
C1—C2—C3—C4	177.9 (5)	C8—C9—C10—N1	−10.4 (4)
C2—C3—C4—C5	−6.1 (7)	N1—N2—C11—C12	−170.6 (3)
C3—C4—C5—C6	6.2 (6)	C8—N2—C11—C12	68.2 (4)
C3—C4—C5—C8	−176.1 (4)	N1—N2—C11—C16	6.1 (5)
C4—C5—C6—C7	−4.2 (6)	C8—N2—C11—C16	−115.1 (3)
C8—C5—C6—C7	177.9 (3)	N2—C11—C12—C13	177.7 (4)
C3—C2—C7—C6	−1.6 (7)	C16—C11—C12—C13	0.9 (6)
C1—C2—C7—C6	−176.2 (4)	C11—C12—C13—C14	−1.1 (6)
C5—C6—C7—C2	2.0 (6)	C12—C13—C14—C15	−0.5 (6)
C4—C5—C8—C9	121.9 (4)	C13—C14—C15—C16	2.3 (7)
C6—C5—C8—C9	−60.5 (4)	C14—C15—C16—C11	−2.4 (6)
C4—C5—C8—N2	2.0 (5)	N2—C11—C16—C15	−175.9 (3)
C6—C5—C8—N2	179.6 (3)	C12—C11—C16—C15	0.8 (5)

### Hydrogen-bond geometry (Å, °)

**Table:**

*D*—H···*A*	*D*—H	H···*A*	*D*···*A*	*D*—H···*A*
OW2—HW2B···OW1	0.85	1.99	2.50 (3)	118
C4—H4A···N2	0.93	2.53	2.875 (6)	102
C7—H7A···F3	0.93	2.41	2.731 (6)	100
C14—H14A···F3^i^	0.93	2.54	3.262 (6)	134
N1—H1A···O^ii^	0.86	1.98	2.811 (6)	161
C9—H9B···O^iii^	0.97	2.60	3.555 (7)	168

Symmetry codes: (i) *x*−1, *y*+1, *z*; (ii) −*x*+1, −*y*+2, −*z*+2; (iii) −*x*+2, −*y*+2, −*z*+2.

## References

[bb1] Allen, F. H., Kennard, O., Watson, D. G., Brammer, L., Orpen, A. G. & Taylor, R. (1987). *J. Chem. Soc. Perkin Trans. 2*, pp. S1–19.

[bb2] Enraf–Nonius (1985). *CAD-4 Software* Enraf–Nonius, Delft, The Netherlands.

[bb3] Harms, K. & Wocadlo, S. (1995). *XCAD4* University of Marburg, Germany.

[bb4] James, D. M. & William, D. B. (2003). WO Patent No. 2003055860.

[bb5] Jia, H.-S., Li, Y.-F., Liu, Y.-Y., Liu, S. & Zhu, H.-J. (2008). *Acta Cryst.* E**64**, o855. 10.1107/S1600536808009823 PMC2961278 21202342

[bb6] Menozzi, G., Mosti, L. & Schenone, P. (1990). *Farmaco*, **45**, 167–186. 2133993

[bb7] North, A. C. T., Phillips, D. C. & Mathews, F. S. (1968). *Acta Cryst.* A**24**, 351–359.

[bb8] Sheldrick, G. M. (2008). *Acta Cryst.* A**64**, 112–122. 10.1107/S0108767307043930 18156677

[bb9] Shi, H., Zhu, H.-J. & Wang, J.-T. (2006). *Acta Cryst.* E**62**, o233–o235.

